# Supplementary material for: Chronic Community Exposure to Environmental Metal Mixtures Is Associated with Selected Cytokines in the Navajo Birth Cohort Study (NBCS)
Source: Int J Environ Res Public Health. 2022 Nov 13;19(22):14939. doi: 10.3390/ijerph192214939 (PMC9690552; doi:10.3390/ijerph192214939)
Supplement: Supplementary file 1 [file ijerph-19-14939-s001.zip › ijerph-2011002-supplementary.pdf]

SUPPLEMENTAL INFORMATION

Table S1. All significant correlations between biomonitored metals, arranged in descending order by Spearman's rho.

Key: THG = Total mercury, BPB = Lead blood, UPB = Lead urine, BCD = Cadmium blood, UCD = Cadmium urine, BMN = Manganese blood, UMN = Manganese urine, UUR = Uranium urine, UTAS = Total arsenic urine, UAS3 = Arsenite (AsIII) urine, UMMA = Monomethylarsonic acid urine, UDMA = Dimethylarsinic acid urine.

Metal 1	Metal 2	rho	p
UDMA	UTAS	0.78	0
UDMA	UMMA	0.67	0
UAS3	UMMA	0.65	0
UAS3	UDMA	0.53	0
BPB	UPB	0.51	0
UAS3	UTAS	0.49	0
UMMA	UTAS	0.48	0
BCD	UCD	0.45	0
UDMA	UMN	0.37	0
UMN	UPB	0.35	0
UDMA	UPB	0.34	0
UPB	UTAS	0.3	0
UPB	UUR	0.29	0
UMMA	UMN	0.29	0
UTAS	UUR	0.26	0
BCD	BPB	0.23	0
UDMA	UUR	0.22	0
UMMA	UPB	0.22	0
UMMA	UUR	0.21	0
BCD	UPB	0.21	0
UAS3	UPB	0.2	0
BMN	BPB	0.19	0.01
UAS3	UMN	0.17	0.01
UAS3	UUR	0.16	0.02

BPB	UUR	0.16	0.02
UMN	UUR	0.16	0.02
UMN	UTAS	0.16	0.02
BPB	UMMA	0.16	0.02
BCD	BMN	0.15	0.03
BMN	UAS3	-0.14	0.05

Table S2. Summary statistics for cytokine measurements (ng/pL)

Cytokine	n	Mean (SD)	Median (IQR)
IL-4	228	0.02 (0.05)	0.01 (0.01-0.02)
IL-6	228	1.11 (1.12)	0.89 (0.54-1.29)
IL-7	228	15.35 (6.01)	14.11 (11.16-18.39)
IL-10	228	0.32 (0.52)	0.2 (0.12-0.32)
IL-12	228	0.09 (0.12)	0.08 (0.03-0.09)
IL-17	228	2.29 (20.48)	0.62 (0.29-1.13)
IL-29	221	0.48 (1.24)	0.12 (0.01-0.62)
IFNα	221	0.61 (1.78)	0.21 (0.05-0.81)
IFNγ	228	9.57 (16.69)	5.62 (3.75-8.35)
TNFα	228	2.04 (1.98)	1.79 (1.43-2.22)
CRP	221	25314.01 (23062.58)	20259.12 (9546-31752)
